# Country data on AMR in Kuwait in the context of community-acquired respiratory tract infections: links between antibiotic susceptibility, local and international antibiotic prescribing guidelines, access to medicine and clinical outcome

**DOI:** 10.1093/jac/dkac220

**Published:** 2022-09-06

**Authors:** Didem Torumkuney, Naser Behbehani, James van Hasselt, Mohamed Hamouda, Nergis Keles

**Affiliations:** GlaxoSmithKline, 980 Great West Road, Brentford, Middlesex TW8 9GS, UK; Department of Medicine, Kuwait University, Kuwait City, Kuwait; GlaxoSmithKline, The Campus, Flushing Meadows, 57 Sloane Street, Bryanston, Gauteng, 2021, South Africa; GlaxoSmithKline, 19th Floor Arenco Tower, Sh. Zayed Road, PO Box 50199, Dubai, UAE; GlaxoSmithKline, Büyükdere Cad. No: 173, 1. Levent Plaza B Blok 34394 Levent, İstanbul, Türkiye

## Abstract

**Background:**

Antimicrobial resistance (AMR) is one of the biggest threats to global public health. Selection of resistant bacteria is driven by inappropriate use of antibiotics, amongst other factors. COVID-19 may have exacerbated AMR due to unnecessary antibiotic prescribing. Country-level knowledge is needed to understand options for action.

**Objectives:**

To review AMR in Kuwait and initiatives underway addressing it. Identifying any areas where more information is required will provide a call to action to minimize any further rise in AMR within Kuwait and to improve patient outcomes.

**Methods:**

National initiatives to address AMR, antibiotic use and prescribing, and availability of susceptibility data, particularly for the key community-acquired respiratory tract infection (CA-RTI) pathogens *Streptococcus pneumoniae* and *Haemophilus influenzae*, were identified. National and international antibiotic prescribing guidelines commonly used locally for specific CA-RTIs (community-acquired pneumonia, acute otitis media and acute bacterial rhinosinusitis) were also reviewed, plus local antibiotic availability. Insights from a clinician in Kuwait were sought to contextualize this information.

**Conclusions:**

In Kuwait there have been some initiatives addressing AMR such as annual campaigns for proper use of antibiotics. Antibiotic use is high but there appears to be a low understanding in the general public about their appropriate use. However, there is legislation in place prohibiting over-the-counter purchase of antibiotics. Only international guidelines for CA-RTIs are used. A more standardized inclusive approach in developing local guidelines, using up-to-date surveillance data of isolates from community-acquired infections in Kuwait, could make management guideline use more locally relevant for clinicians. This would pave the way for a higher level of appropriate antibiotic prescribing and improved adherence. This would, in turn, potentially limit AMR development and improve clinical patient outcomes.

## Introduction

Antimicrobial resistance (AMR) is one of the biggest threats to public health throughout the world^1^ as described in the introductory paper of this Supplement.^2^ The WHO states that ‘the world urgently needs to change the way it prescribes and uses antibiotics. Even if new medicines are developed, without behaviour change, antibiotic resistance will remain a major threat’.^3^ The first paper in this Supplement included details about the multiple factors which can drive a rise in AMR along with the global initiatives that are in place to address this threat.^2^ Each country and/or region must also play their part through local initiatives.

In order to identify how AMR can be addressed in Kuwait in the future, it is necessary to review what is happening now. In this paper, we present the current situation in Kuwait, determined by using published information (from searching PubMed, Google Scholar and other internet platforms) to ascertain any national initiatives to address AMR, antibiotic use and prescribing, and availability of susceptibility data, in particular for the key community-acquired respiratory tract infection (CA-RTI) pathogens *Streptococcus pneumoniae* and *Haemophilus influenzae*. National and international antibiotic prescribing guidelines for CA-RTIs, specifically community-acquired pneumonia (CAP), acute otitis media (AOM) and acute bacterial rhinosinusitis (ABRS), commonly used by healthcare professionals in Kuwait were also reviewed, along with how these link to local antibiotic availability. Insights from a clinician in Kuwait were sought to contextualize this information. In addition, we aimed to identify areas where more information is required and present a call to action to improve clinical outcome for patients and to minimize further rises in AMR within Kuwait.

## Action Plans

Following the formulation by the World Health Assembly in 2015 of a Global Action Plan (GAP) for AMR^4^ many countries began to develop their own National Action Plan (NAP) and in 2018, the WHO reported good progress in the Eastern Mediterranean region in the development of NAPs on AMR. Ten countries had submitted or completed plans and a further seven were in the process of producing plans, including Kuwait,^5^ although the current NAP status, as reported by the WHO for 2020–21, shows that Kuwait had yet to finalize an AMR NAP.^6^

## Antibiotic prescribing and use

In Kuwait and the Gulf Cooperation Council countries, inappropriate prescribing of antibiotics and self-medication are widespread risk factors for increasing antibiotic resistance. A lack of policies available for limiting inappropriate antibiotic use contributes to the increase in AMR in the region. Although progress is being made,^7^ the number of antimicrobial stewardship programmes is low in Kuwait.^8–10^

A study exploring the patterns of antibiotic resistance found that Kuwait had a growth rate of resistance of 17% for several common pathogens between 1999 and 2003.^11^ Another study on resistance trends in *S. pneumoniae* in a tertiary hospital in Kuwait during 1997–2007, clearly demonstrated an increase in the prevalence of AMR and drug resistant *S. pneumoniae*.^12^

The use of antibiotics in treating upper respiratory tract infections (URTIs) was evaluated in primary healthcare centres in Kuwait and the extent to which antibiotic use followed international guidelines was assessed. Half of all URTIs were treated with antibiotics, but of these, 94% (127/135 prescriptions) could not be justified when applying the UK National Institute for Health and Care Excellence (NICE) guidelines that were current at the time of the study. This overuse of antibiotics was linked primarily to the lack of evidence-based practice in Kuwait.^13^

Knowledge, attitudes and practices regarding antibiotic use amongst the public was investigated in a study of 770 randomly selected Kuwaiti individuals.^14^ 73% of the respondents had been prescribed an antibiotic in the last 12 months and, of these, 36% failed to finish the course, mainly because they felt better. Antibiotics were used without a medical consultation by 27.5% of participants (187), primarily to treat the common cold, sore throat or cough. There was confusion about whether antibiotics were effective against bacteria or viruses, and almost 47% had little knowledge about the action of, safety and resistance to antibiotics.^14^

Further work is needed to build awareness of the appropriate use of antibiotics by the public in Kuwait. Studies suggest a multidisciplinary approach must be put in place to: educate the public on appropriate antibiotic use; improve policies regarding the rational prescription of antimicrobials; and increase regulatory control.^15^

## Surveillance

### Global surveillance studies

#### SOAR

Several ongoing global surveillance studies provide antibiotic susceptibility data from Kuwait. The Survey of Antibiotic Resistance (SOAR), a multinational antibiotic surveillance study, has been underway in an expanding range of countries since 2002. The study aims to collect and make available in published, peer-reviewed papers, antibiotic susceptibility data, specifically for *S. pneumoniae* and *H. influenzae*, which are the most commonly isolated respiratory pathogens in the community.^16^ Key features of the SOAR study are that it focuses on these pathogens only, and that identification and susceptibility testing are performed in an independent centralized laboratory using standardized methodology (CLSI) allowing for comparisons to be made between countries or regions and for the identification of trends over time. SOAR data is analysed based on three different breakpoints: CLSI, EUCAST dose-specific and PK/PD breakpoints.

Clinical breakpoints are cut-off MIC values used to classify microorganisms into the clinical categories susceptible (S), intermediate (I) and resistant (R) in order to assist in the prediction of the clinical success or failure of a specific antibiotic.^17^ CLSI and EUCAST define breakpoint values but due to variation in criteria for their definition, there are some differences between CLSI and EUCAST in the clinical breakpoint values for certain bacteria for some antibiotics and this can impact susceptibility interpretation of clinical isolates.^18^ EUCAST breakpoints are dose-specific and use the EMA-approved doses that are included in the Summary of Product Characteristics of an antibiotic. This means that by application of breakpoints for higher doses, the effect of using a raised dose on the clinical efficacy of a particular antibiotic can be predicted. Currently, clinical microbiology laboratories in Kuwait use CLSI breakpoints, however the international application of the EUCAST breakpoints is expanding,^19^ so it is possible that dose-specific breakpoints could also be applied in Kuwait in the future. The EUCAST dose-specific breakpoints can also be used retrospectively to calculate the susceptibility of previously collected isolates to show susceptibility levels that would have been achievable with higher doses.

Use of the EUCAST dose-specific breakpoints shows the effect of increasing the antibiotic dose on the susceptibility of a pathogen, providing additional information so that the prescriber can decide whether a higher dose would be of benefit. For example, *S. pneumoniae,* the most commonly isolated respiratory pathogen,^20,21^ causing conditions such as CAP, AOM, and ABRS, has over time become less susceptible to amoxicillin/clavulanic acid in some countries^22^ since the MIC of some isolates has increased. When treating infections, it is important to be able to eradicate bacterial pathogens with raised MICs to optimize clinical outcome while at the same time minimizing the risk of selecting variants with even higher MICs. This is possible because β-lactams, unlike many other antibiotics, have time-dependent killing properties. Their efficacy depends on the amount of time the drug concentration is present at the site of action; the use of higher doses and/or more frequent dosing allows for successful eradication of pathogens with higher MICs because the time above the MIC is increased.^23^

**Figure 1.**
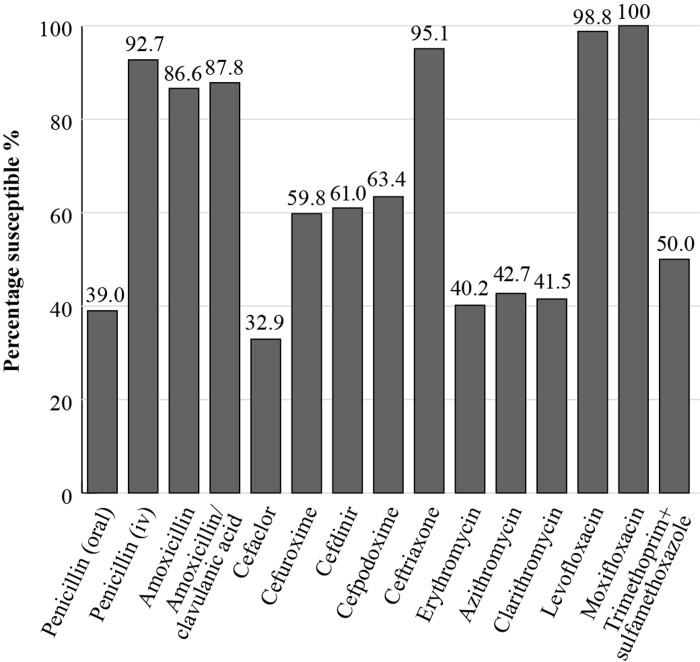
Percentage susceptibility rates based on CLSI breakpoints for antibiotics against *S. pneumoniae* isolates (*n *= 82) collected as part of the SOAR study in Kuwait in 2015–17.

The most recent SOAR results for Kuwait were obtained from a single centre, Kuwait University. Clinical isolates were collected from patients with CA-RTIs. When applying CLSI breakpoints, susceptibility of *S. pneumoniae* isolates (*n *= 82) to amoxicillin and amoxicillin/clavulanic acid was 86.6% and 87.8%, respectively (Figure 1). Notably, lower susceptibility to the macrolides was observed for *S. pneumoniae* isolates (erythromycin 40.2%; clarithromycin 41.5% and azithromycin 42.7%).^16^

#### ATLAS

As outlined in the introductory paper to this Supplement,^2^ the Antimicrobial Testing Leadership and Surveillance (ATLAS) database is fully accessible and searchable and covers susceptibilities of a range of bacterial and fungal pathogens to a bank of antimicrobials.^24^

**Figure 2.**
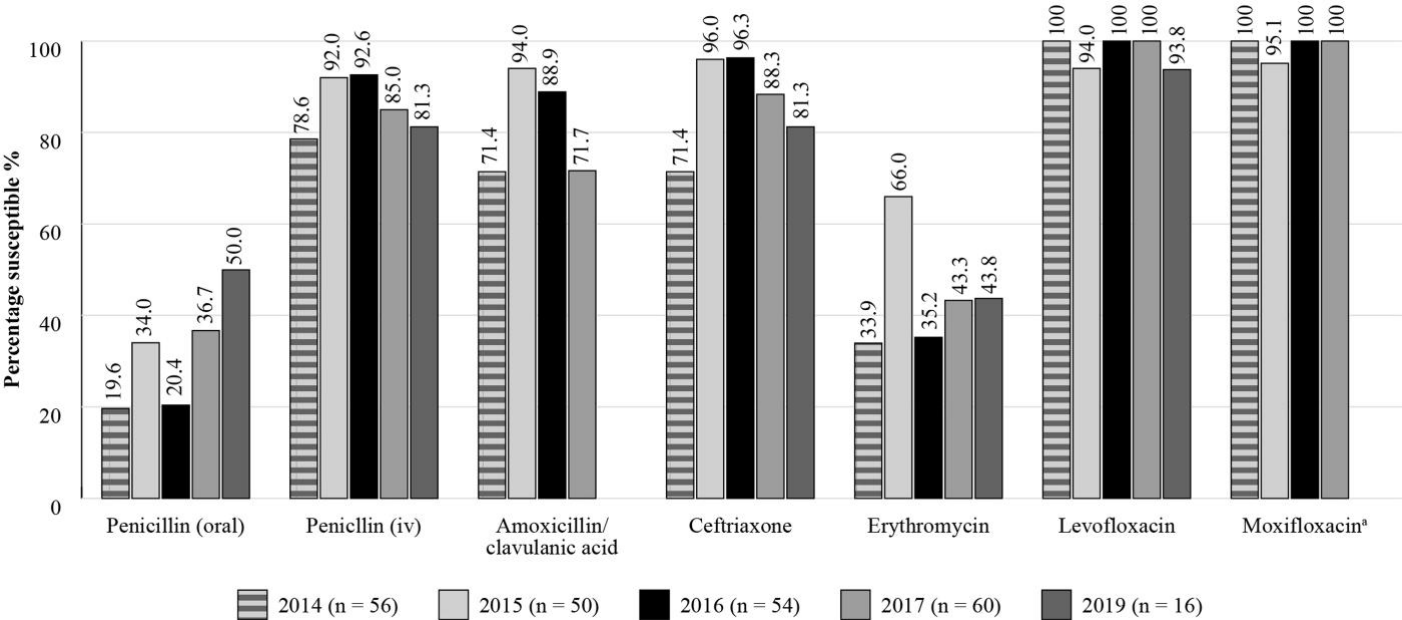
Percentage susceptibility rates based on CLSI breakpoints for antibiotics against *S. pneumoniae* isolates from the ATLAS surveillance programme in Kuwait in 2014–19. Data access date 21 November 2021.

**Figure 3.**
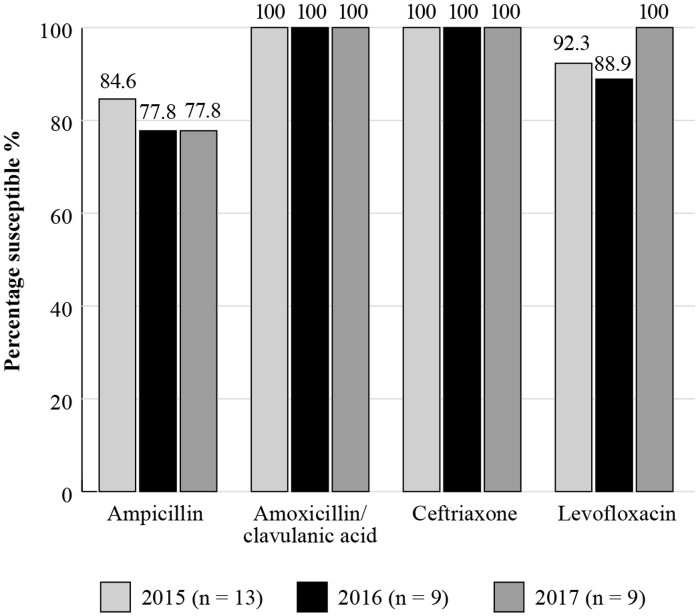
Percentage susceptibility rates based on CLSI breakpoints for antibiotics against *H. influenzae* isolates from the ATLAS surveillance programme in Kuwait 2015–17. Data access date 21 November 2021.

Results for *S. pneumoniae* are shown in Figure 2. The susceptibility of *S. pneumoniae* isolates from outpatients in Kuwait with RTIs to amoxicillin/clavulanic acid ranged from 71.4% to 94.0% between 2014 and 2019. Susceptibility to the macrolide erythromycin (which can be used to predict the susceptibility of azithromycin and clarithromycin based on CLSI guidelines), remained low (33.9%–66.0%) over this entire period. A small number of *H. influenzae* isolates was collected between 2015 and 2017 and showed high susceptibility (88.9%–100%) to the antibiotics tested, including amoxicillin/clavulanic acid (100%), apart from a small reduction in the susceptibility to ampicillin (77.8%–84.6%) (Figure 3). ATLAS data is analysed based on CLSI and EUCAST breakpoints.

#### GLASS

In 2015, WHO launched the Global Antimicrobial Resistance and Use Surveillance System (GLASS). GLASS is a global system which collects national AMR data for selected bacterial pathogens that cause common infections. The aim is to monitor the prevalence of AMR amongst major pathogens in clinical settings^25^ to provide supporting data required to ensure that countries can design cost-effective, evidence-based AMR response strategies. During the first four years, 91 countries/territories have enrolled in GLASS and data for over two million patients from 66 countries are included.^26^

Pathogens currently included in GLASS-AMR are: *Acinetobacter* spp., *Escherichia coli*, *Klebsiella pneumoniae*, *Neisseria gonorrhoeae*, *Salmonella* spp., *Shigella* spp., *Staphylococcus aureus*, and *S. pneumoniae*. By 2021, the number of countries to have enrolled in GLASS had increased to 107, including Kuwait.^27^ An important new component in GLASS is the inclusion of antimicrobial consumption (AMC) surveillance at the country, regional and global level and the aim is to provide data on the use of antimicrobials in the population. GLASS data is analysed based on CLSI and EUCAST breakpoints.

## Disease Management Guidelines

Most guidelines suggest a first-line antibiotic or antibiotics along with alternatives and then a second-line antibiotic or antibiotics, also with alternatives. The first-line antibiotic is the recommended initial choice that should be prescribed by the clinician following diagnosis of the infection, supported by the criteria defined by the organization or committee; alternatives may be provided for use in particular circumstances. The second-line antibiotic is for use if the first-line antibiotic does not achieve the anticipated outcome, and again alternatives may be included for specific circumstances.

For management of the common RTIs, CAP, AOM and ABRS in Kuwait, clinicians make use of a range of international guidelines, examples of which are shown in Table 1.

**Table 1.** Examples of international antibiotic prescribing guidelines referred to by physicians in Kuwait for the management of community-acquired respiratory tract infections

International antibiotic prescribing guidelines
IDSA 2007: Infectious Diseases Society of America/American Thoracic Society consensus. guidelines on the management of community-acquired pneumonia in adults^28^
BTS 2009: British Thoracic Society guidelines for the management of community-acquired pneumonia in adults: update 2009^29^
IDSA 2011 (Endorsed by AAP): The management of community-acquired pneumonia in infants and children older than 3 months of age: clinical practice guidelines by the Pediatric Infectious Diseases Society and the Infectious Diseases Society of America^30^
BTS 2011: British Thoracic Society guidelines for the management of community-acquired pneumonia in children: update^31^
IDSA 2012: IDSA Clinical practice guideline for acute bacterial rhinosinusitis in children and adults^32^
AAP 2013: American Academy of Pediatrics. The diagnosis and management of acute otitis media^33^
IDSA 2019: Diagnosis and treatment of adults with community-acquired pneumonia. An official clinical practice guideline of the American Thoracic Society and Infectious Diseases Society of America^34^

### International antibiotic prescribing guidelines

For the management of CAP in adults and paediatrics, the international guidelines referred to by clinicians in Kuwait include those from the British Thoracic Society (BTS)^31^ and the IDSA for adults^34^ and children.^30^ For example, a first-line antibiotic treatment recommendation for CAP management from the IDSA 2019 guideline for treating adults with no comorbidities or risk factors for MRSA or *Pseudomonas aeruginosa*, is amoxicillin or doxycycline or a macrolide (if the local pneumococcal resistance is <25%) but if the patient has comorbidities, the recommendation is combination therapy with amoxicillin/clavulanic acid or a cephalosporin plus a macrolide or doxycycline, or monotherapy with a respiratory fluoroquinolone. The recommended dosage for adults with comorbidities is amoxicillin/clavulanic acid, 500 mg/125 mg given three times daily, 875 mg/125 mg or 2000 mg/125 mg both given twice daily in combination with a macrolide or doxycycline.^34^ The BTS also suggests amoxicillin/clavulanic acid, cefaclor, and macrolides as an alternative to first-line amoxicillin in children with low-severity pneumonia.

For the management of AOM, the international guidelines referred to in Kuwait are principally those from the American Academy of Pediatrics (AAP).^33^ In the AAP guidelines, for initial or delayed treatment amoxicillin or amoxicillin/clavulanic acid are recommended. In the case of penicillin allergy, alternatives are cefdinir, cefuroxime, cefpodoxime or ceftriaxone. If there is failure of initial treatment then ceftriaxone or amoxicillin/clavulanic acid is recommended. For the management of ABRS in adults and children the international guidelines used include those from the IDSA,^32^ which recommend amoxicillin/clavulanic acid rather than amoxicillin alone as initial empirical treatment.

### National antibiotic prescribing guidelines

Currently there are no local guidelines available or recently published for the management of CAP, AOM and ABRS in Kuwait apart from guidelines published for the management of pneumonia in COVID-19 patients.

## Antibiotic availability

In Kuwait, several currently available formulations of amoxicillin/clavulanic acid are mentioned as first- or second-line recommendations by international RTI management guidelines. This includes the IDSA^34^ where for outpatients with comorbidities and CAP, the higher dose regimens of amoxicillin/clavulanic acid 875 mg/125 mg or 2000 mg/125 mg both given twice daily are recommended in addition to 500 mg/125 mg three times daily, for use in combination with a macrolide or doxycycline. In children who are outpatients with presumed pneumonia, amoxicillin/clavulanic acid (amoxicillin component, 90 mg/kg/day twice daily) is recommended by the IDSA as an alternative empirical therapy to first-line amoxicillin treatment for CAP.^30^

In adults with ABRS, the IDSA guideline 2012^32^ recommends either 500 mg/125 mg three times daily or 875 mg/125 mg twice daily as initial first-line therapy. In children with no β-lactam allergy, the IDSA suggests that first-line initial empirical therapy in ABRS is amoxicillin/clavulanic acid 45 mg/kg/day given twice daily. High dose regimens are recommended where there is risk of antibiotic resistance (specifically endemic penicillin-non-susceptible *S. pneumoniae)* or for failed initial empirical therapy, the 90 mg/kg/day regimen (based on amoxicillin component) given twice daily is recommended for children and 2 g/day given twice daily for adults.

## Quality of medicines

Substandard poor-quality or falsified antibiotics promote AMR and the spread of drug-resistant infections. Since poor-quality antibiotics are unlikely to contain the full dose needed to eliminate all infecting pathogens, this encourages resistance to develop and allow resistant strains to survive and be transmitted.^35^

The quality of medicines, specifically antibiotics, is an important consideration for countries worldwide. WHO launched a Global Surveillance and Monitoring System (GSMS) for substandard and falsified products.^35^ The GSMS aims to work with WHO member states to improve the quality of reporting of substandard and falsified medical products, and, importantly, to ensure the data collected are analysed and used to influence policies, procedures, and processes to protect public health at all levels. Use of substandard or falsified antibiotics not only compromises clinical outcome but also risks increased AMR. The most recent summary (2013–17) reported substandard and falsified medicines in 46 member states. Antibiotics represented 16.9% of all products reported, second only to malaria drugs (19.6%).

## Local insight

### Clinician expert comment

Kuwait, like many other countries, has a problem of increasing AMR amongst several common bacteria that cause lower RTIs as documented by recent studies.^16,36^ This poses an alarming trend and represents a genuine threat to the health of the population and availability of effective antibiotics. Large hospitals in Kuwait publish antibiograms, which outline the resistance pattern of nearly all organisms isolated in that hospital. The microbiology department usually holds a meeting with the department concerned to highlight these resistance patterns and their implications for the proper use of antibiotics in identified infections. However, more effort is needed to make use of these antibiograms and to construct hospital-based antibiotic policies.

Kuwait, unlike some other countries in The Gulf or Arab region, does not allow over-the-counter (OTC) dispensing of antibiotics, although this does happen to a certain extent. However, there is overuse of antibiotics in public health institutions and more so in private health facilities.

There are several attempts and efforts to try to control the problems of infections in general and overuse of antibiotics, in particular. The Kuwait Ministry of Health has a designated Infection Control Directorate which deals mainly with efforts to control health facility-related infections.^37^ The infection control directorate also runs an annual campaign for proper use of antibiotics.^38^ As far as antibiotics policy is concerned there are, unfortunately, no well-publicized or well-used national guidelines/policies. There are some hospital-based policies but often the selection of antibiotics is dictated by physician choice rather than health facility policy. The eight Consensus Principles^39,40^ to address increasing antibiotic resistance represent an ideal framework. The challenge for each country is, however, how to implement these principles by using national guidelines that are adhered to, audits, and monitoring of antibiotic usage across all health facilities.

## Conclusions

In an era of rising AMR globally, this paper aims to define areas where action is required to address AMR by analysing and understanding the current situation within a specific country. Information is presented for Kuwait concerning antibiotic use and prescribing, approach to AMR, availability of local susceptibility data, use of international and/or local antibiotic prescribing guidelines and how these link to antibiotic availability. To our knowledge this is the first time this information has been reviewed and presented in detail by country.

Whilst the WHO has produced a GAP on AMR, this has yet to be followed up in Kuwait with production of a NAP and, as explained by the clinician from Kuwait in this paper, in terms of national antibiotics policy there are no well-publicized or well-used national guidelines.

Inappropriate antibiotic prescribing in the Gulf Region is widespread and is associated with antibiotic resistance and is leading to multidrug resistance and ultimately an increased mortality rate. The lack of policies available for limiting inappropriate antibiotic use contributes to the increase in antibiotic resistance in the region.^17,19^ Whilst laws exist prohibiting OTC purchase of antibiotics, the clinician’s comments in this paper indicate that this does still happen. In Kuwait, studies have reported overuse of antibiotics in RTIs and a widespread poor understanding by members of the public of the appropriate use, safety and resistance to antibiotics.

In terms of surveillance, the global surveillance studies ATLAS and SOAR include data for Kuwait on the susceptibility of CA-RTI pathogens and reveal a trend towards decreasing susceptibility amongst the common isolates for some antibiotic classes, such as the macrolides. The fluoroquinolone antibiotics have so far retained activity, although guidelines and regulatory bodies urge caution, restricting their use to limited situations due to serious safety concerns.

For management of the common RTIs in Kuwait, clinicians make use of international antibiotic prescribing guidelines and for the management of CA-RTIs, amoxicillin/clavulanic acid is a commonly recommended antibiotic, where its choice is substantiated by the available susceptibility data.

As mentioned in the clinician’s comments, large hospitals maintain antibiograms of susceptibilities which can be applied to produce an antibiotic policy within that hospital, but an increase in local surveillance studies within the community would lead to the development of regularly updated management guidelines. These, if applied along with updated guidelines relating to the management of COVID-19 patients, would improve clinical outcomes and minimize further rises in AMR.

While a range of international guidelines is utilized by clinicians in Kuwait, a more standardized inclusive approach is needed to develop local country-specific guidelines These guidelines should be based on up-to-date surveillance data of isolates from community-acquired infections, which would make them more locally relevant for clinicians, reiterating the Consensus Principles as described in the introductory paper to this Supplement.^2^ This would pave the way for improved adherence and a higher level of appropriate antibiotic prescribing in CA-RTIs which could, in turn, potentially limit AMR development and improve clinical outcomes for patients.
